# Comparison of the Prognostic Performance of the Bedside Index for Severity in Acute Pancreatitis (BISAP) and Emergency Room Assessment of Acute Pancreatitis (ERAP) Scores in Acute Pancreatitis

**DOI:** 10.7759/cureus.98840

**Published:** 2025-12-09

**Authors:** Sri Hari Babu Sunkari, Siddharth P Dubhashi, Ajay A

**Affiliations:** 1 Trauma and Emergency, All India Institute of Medical Sciences Nagpur, Nagpur, IND; 2 General Surgery, All India Institute of Medical Sciences Nagpur, Nagpur, IND

**Keywords:** acute pancreatitis, bisap, erap, mortality, organ failure, severity

## Abstract

Background: Early identification of severe acute pancreatitis (AP) is crucial for timely intervention, optimal resource utilization, and better outcomes. Although several scoring systems exist, their accuracy and applicability in emergency settings remain uncertain. This study evaluated the prognostic accuracy of the Emergency Room Assessment of Acute Pancreatitis (ERAP) score and compared it with the Bedside Index for Severity in Acute Pancreatitis (BISAP) score for predicting severe AP, mortality, and organ failure in an Indian population and emergency settings.

Materials and methods: We conducted a prospective observational study in the Department of Trauma and Emergency at All India Institute of Medical Sciences (AIIMS), Nagpur, between December 2023 and January 2025. Patients over 18 years old diagnosed with AP using the Revised Atlanta Criteria were included. Admission data were used to calculate ERAP and BISAP scores. Predictive accuracy for severe AP, mortality, and organ failure was assessed via the area under the curve (AUC).

Results: Among 165 patients (mean age 37.9 ± 12.1 years), alcohol (139 (84.2%)) was the predominant cause for AP. Severe AP occurred in 31 (18.8%). The ERAP and BISAP scores showed AUCs of 0.852 vs. 0.784 for severe AP, 0.853 vs. 0.778 for mortality, and 0.843 vs. 0.844 for any organ failure, with an optimal cutoff ≥ 2 for both. ERAP demonstrated numerically higher prognostic accuracy than BISAP for severe AP (*P* = 0.1190) and mortality (*P* = 0.2829), but those were not statistically significant.

Conclusions: ERAP showed slightly higher but statistically insignificant predictive accuracy than BISAP, suggesting ERAP as an alternative for early risk stratification in emergency settings.

## Introduction

Acute pancreatitis (AP) is a potentially life-threatening inflammatory condition of the pancreas that frequently presents as a medical emergency. The global incidence of AP has been increasing steadily, with reported rates ranging between 13 and 45 per 100,000 population annually, making it a significant clinical and public health concern [1]. In tertiary care hospitals across India, AP contributes substantially to emergency admissions for abdominal pain, with approximately 10%-30% of cases progressing to moderate or severe forms requiring intensive care and increased resource utilization [2].

Accurate early risk stratification in AP is essential for guiding timely interventions, determining the level of care, and improving outcomes. Several clinical scoring systems have been proposed for this purpose. However, many of them, such as Ranson’s criteria [3] and the Glasgow-Imrie score [4], depend on variables measured over 48 hours, making them less practical for early use in emergency settings. The Acute Physiology and Chronic Health Evaluation (APACHE) II score [5] can be applied at admission but is complex and non-specific, limiting its feasibility in fast-paced environments like the emergency department (ED).

The Bedside Index for Severity in Acute Pancreatitis (BISAP) score [6] was developed to predict disease severity using five easily available clinical and laboratory parameters: blood urea nitrogen (BUN), age, systemic inflammatory response syndrome (SIRS), altered mental status, and pleural effusion. Although BISAP is widely used and validated, it has limitations, especially among younger patients, and does not assess local pancreatic or peripancreatic complications [7].

To address these limitations, newer, ED-oriented tools have been developed, such as the Emergency Room Assessment of Acute Pancreatitis (ERAP) score [8]. ERAP incorporates respiratory rate, Glasgow Coma Scale, C-reactive protein (CRP), and BUN levels, enabling rapid bedside assessment at presentation. Preliminary research suggests that ERAP may accurately predict severe AP, organ failure, and mortality in the emergency setting. Hence, this study aimed to evaluate the prognostic accuracy of ERAP and compare it with BISAP in predicting severe AP, mortality, and organ failure among patients presenting to the ED in an Indian population.

## Materials and methods

Study design and setting

This prospective observational study was conducted in the Department of Trauma and Emergency, All India Institute of Medical Sciences (AIIMS) Nagpur between December 2023 and January 2025. Written informed consent was obtained from all participants, and patient information sheets were provided.

Study population

All adult patients (≥18 years) presenting to the ED with acute abdominal pain and subsequently diagnosed with AP were screened for inclusion. Diagnosis was based on the Revised Atlanta Classification 2012 [9], requiring at least two of the following: (1) abdominal pain characteristic of AP, (2) serum amylase or lipase ≥3 times the upper limit of normal, and (3) characteristic findings of AP on imaging (CT, MRI, or ultrasound). Exclusion criteria included chronic pancreatitis, AP treated outside the institution for any complication, psychiatric illness, and patients unwilling to participate.

Data collection

Demographic details, clinical features, vital signs, laboratory parameters, and imaging findings were recorded at admission using a standardized proforma. All patients underwent contrast-enhanced CT as part of routine evaluation. The variables used to calculate the ERAP and BISAP scores were recorded prospectively at admission as per their original definitions. The BISAP score ranges from 0 to 5, with one point assigned for each of the following five criteria: BUN > 25 mg/dL, impaired mental status (Glasgow Coma Scale < 15), presence of SIRS, age > 60 years, and the presence of pleural effusion on imaging. A score of more than 2 is considered severe AP, and a score of less than or equal to 2 is considered non-severe AP, which includes both mild and moderately severe AP. The ERAP score ranges from 0 to 4, assigning one point for each of the following four criteria: respiratory rate 22 breaths/minute, Glasgow Coma Score < 15, BUN 25 mg/dL, and CRP level > 150 mg/L. An ERAP score of 2 or greater is considered severe AP, and a score of less than or equal to 2 is considered non-severe AP.

Outcome measures

The primary outcome was disease severity (mild, moderate, or severe) per the Revised Atlanta Criteria 2012 [9]. Organ failure was assessed using the modified Marshall scoring system at 48 hours and categorized as transient or persistent. Secondary outcomes included in-hospital mortality and local or systemic complications. Management followed institutional protocols aligned with the Revised Atlanta Classification 2012 [9] and the 2019 World Society of Emergency Surgery (WSES) guidelines [10].

Statistical analysis

Data were analyzed using STATA version 16 (StataCorp LLC, College Station, TX, US). Distribution of continuous variables was assessed using the Shapiro-Wilk test and visual inspection of histograms. Normally distributed variables were expressed as mean ± SD and compared using the unpaired t-test. Skewed continuous variables were expressed as median (interquartile range (IQR)) and compared using the Mann-Whitney U test. Categorical variables were compared using the Chi-squared test or Fisher’s exact test as appropriate.

Cutoff values were determined using the Youden index. Sensitivity, specificity, positive predictive value (PPV), negative predictive value (NPV), and likelihood ratios (LR) were calculated for both ERAP and BISAP scores. Receiver operating characteristic (ROC) curves were plotted, and area under the curve (AUC) values were compared using the DeLong test. Statistical significance was defined as P < 0.05. The existing ERAP and BISAP scores have been validated for their prognostic performance in predicting disease severity, organ failure, and mortality in the ED setting.

Ethics statement

Written informed consent was obtained from all participants, in accordance with the Declaration of Helsinki. The study was approved by the Institutional Ethics Committee, AIIMS Nagpur (IEC/Pharmac/2023/689).

## Results

Patient characteristics

**Figure 1:**
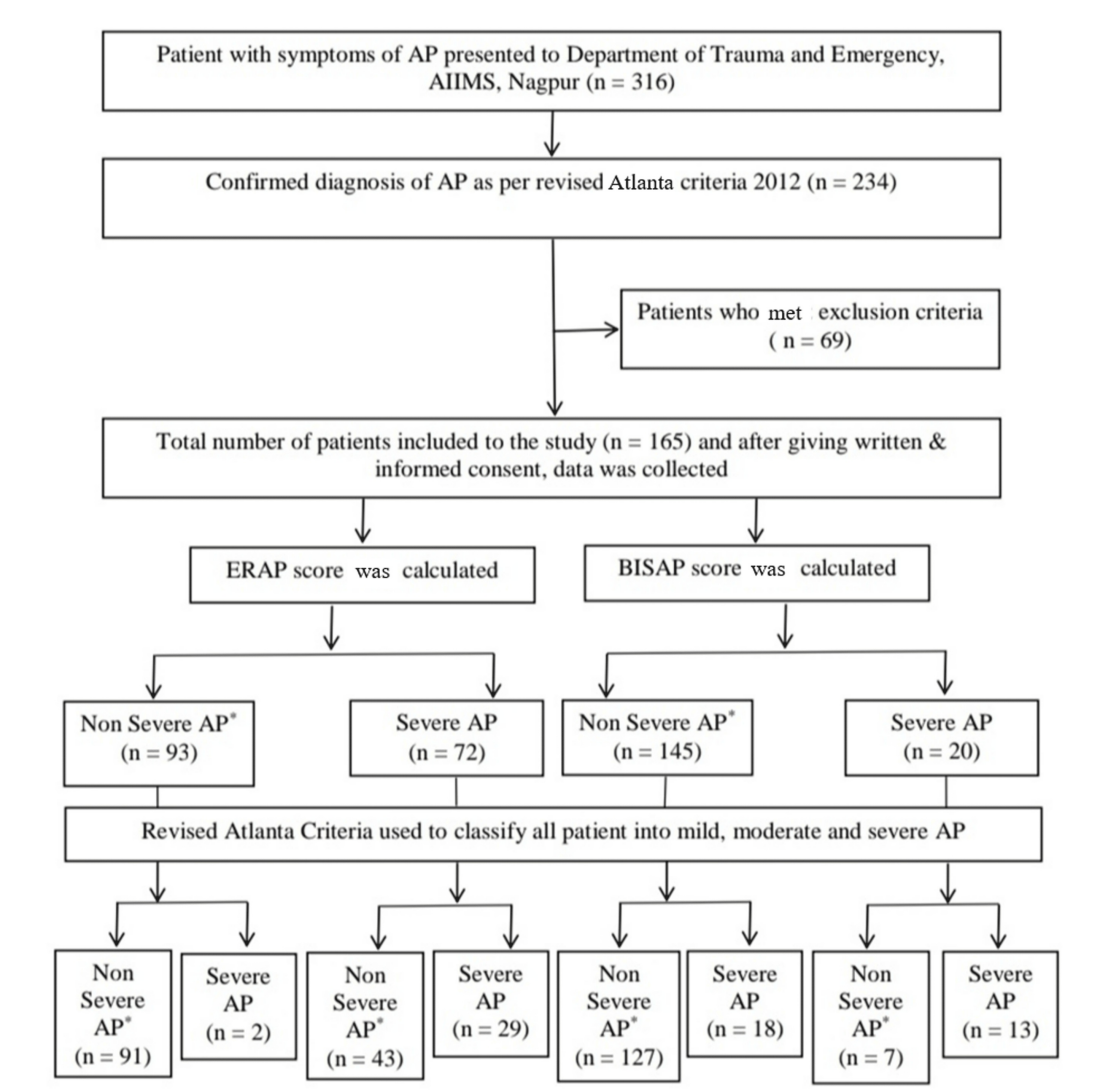
STARD 2015 flow diagram. AP: acute pancreatitis; BISAP: Bedside Index for Severity in Acute Pancreatitis; ERAP: Emergency Room Assessment of Acute Pancreatitis; STARD: Standards for Reporting Diagnostic Accuracy Studies; AIIMS: All India Institute of Medical Sciences. *Non-severe AP include both mild and moderately severe AP. As per Revised Atlanta 2012 guidelines, mild and moderately severe AP cases were grouped together under the “non-severe” category for analytical purposes, while severe AP formed the “severe” group.

From December 2023 to January 2025, 165 patients diagnosed with AP were enrolled (Figure 1).

Baseline demographics and clinical characteristics are summarized in Table 1. The mean age was 37.95 ± 12.11 years, with a male-to-female ratio of 10:1. The predominant etiologies included alcohol (139 (84.2%)), gallstones (14 (8.5%)), and others (12 (7.3%)). Based on the Revised Atlanta 2012 classification, 134 (81.2%) of patients had non-severe AP, including both mild AP and moderately severe AP, and 31 (18.8%) had severe AP with persistent organ failure. Organ failure, both transient and persistent, occurred in 43 (26.1%) of patients, and the overall in-hospital mortality rate was 14 (8.5%), with all deaths occurring in severe AP patients. The median hospital stay was 10.26 ± 9.69 days. The median ERAP and BISAP scores were both 1.

**Table 1:** Clinical and laboratory characteristics. Data represented as mean ± SD, n (%), or median (IQR). HR: heart rate; RR: respiratory rate; SpO_2_: oxygen saturation; Temp: temperature; GCS: Glasgow Coma Scale; BUN: blood urea nitrogen; CRP: C-reactive protein; IQR: interquartile range; AP: acute pancreatitis; TLC: total leukocyte count. Statistical tests used include the unpaired t-test for normally distributed continuous variables and the Mann-Whitney U test for skewed continuous variables. P-values < 0.05 are considered statistically significant. P-values of male sex, alcohol etiology, temperature, lipase, and amylase are not significant.

Variable	Overall (n = 165)	Non-severe AP (n = 134)	Severe AP (n = 31)	Test	Test statistic	P-value
Age (years)	37.9 ± 12.1	36.4 ± 11.9	43.8 ± 11.2	Unpaired t-test	t = 2.56	0.011
Male sex n (%)	150 (90.9)	121 (90.3)	29 (93.5)	Mann-Whitney U	U = 0.20	0.654
Alcohol etiology n (%)	139 (84.2)	113 (84.3)	26 (83.9)	Mann-Whitney U	U = 0.00	0.964
HR (bpm)	100.7 ± 20.1	98.3 ± 19.2	111.3 ± 20.7	Unpaired t-test	t = 2.74	0.007
SpO_2_ (%)	96.6 ± 5.6	97.4 ± 4.8	93.2 ± 7.1	Unpaired t-test	t = 3.19	0.002
RR (breaths/min)	22.8 ± 4.7	22.1 ± 4.4	25.6 ± 4.5	Mann-Whitney U	U = 1,267.0	<0.001
Temperature (°C)	37.3 ± 0.8	37.2 ± 0.7	37.6 ± 0.9	Mann-Whitney U	U = 1,768.5	0.062
GCS	14 (13–15)	15 (14–15)	13 (12–14)	Mann-Whitney U	U = 1,320.5	<0.001
Pain score	6 (4–8)	6 (4–7)	8 (6–9)	Mann-Whitney U	U = 1,739.0	0.031
TLC (×10⁹/L)	11.2 ± 4.8	10.7 ± 4.6	13.4 ± 5.2	Mann-Whitney U	U = 1,522.0	0.012
BUN (mg/dL)	17.0 ± 17.4	14.5 ± 14.8	27.8 ± 22.4	Mann-Whitney U	U = 1,484.0	0.005
CRP (mg/L)	111.9 ± 138.8	93.6 ± 125.2	191.4 ± 165.5	Mann-Whitney U	U = 1,538.0	0.018
Lipase (U/L)	764 (392–1,320)	713 (372–1,220)	980 (520–1,570)	Mann-Whitney U	U = 1,837.0	0.143
Amylase (U/L)	524 (286–842)	496 (278–794)	634 (350–1,018)	Mann-Whitney U	U = 1,885.0	0.224

Characteristics of the ERAP and BISAP scores

Among the four ERAP components, elevated CRP > 150 mg/L was seen in 101 (61.2%) patients, followed by respiratory rate ≥ 22/min in 97 (58.8%). Most patients had an ERAP score of 1 (56 (33.9%)); a score of 3 was less common (12 (7.3%)), and none had 4. Increasing ERAP scores correlated significantly with greater severity, mortality, and organ failure.

Among BISAP components, SIRS ≥ 2 occurred in 103 (62.4%) patients, followed by pleural effusion (61 (36.9%)). Most patients had a BISAP score of 1 (55 (33.3%)), while none scored 4 or 5. A higher BISAP score was similarly associated with worse outcomes.

Using the Youden index, an ERAP score of 2 was identified as the optimal cutoff for predicting severe AP, mortality, and organ failure, demonstrating high sensitivity, specificity, PPV, and NPV, positive LR, and negative LR. Similarly, the optimal BISAP cutoff was identified as 2 for predicting severe AP, mortality, and organ failure (Table 2).

**Table 2:** Diagnostic performance of BISAP and ERAP scores at established cutoff points in predicting severe AP, mortality, and organ failure. Data represented as percent (%). AP: acute pancreatitis; BISAP: Bedside Index for Severity in Acute Pancreatitis; ERAP: Emergency Room Assessment of Acute Pancreatitis; NPV: negative predictive value; PPV: positive predictive value; LR: likelihood ratio. *Includes both transient and persistent organ failure.

	Severe AP	Mortality	Any organ failure*
ERAP			
Cutoff	2	2	2
Sensitivity	93.5	100.0	88.4
Specificity	67.9	61.6	72.1
PPV	40.3	19.4	52.8
NPV	97.8	100.0	94.6
Positive LR	2.92	2.60	3.17
Negative LR	0.10	0.00	0.16
BISAP			
Cutoff	2	2	2
Sensitivity	77.4	78.6	81.4
Specificity	69.4	64.2	75.4
PPV	36.9	16.9	53.8
NPV	93.0	97.0	92.0
Positive LR	2.53	2.20	3.31
Negative LR	0.33	0.33	0.25

Comparative analysis of ERAP and BISAP scores

**Figure 2:**
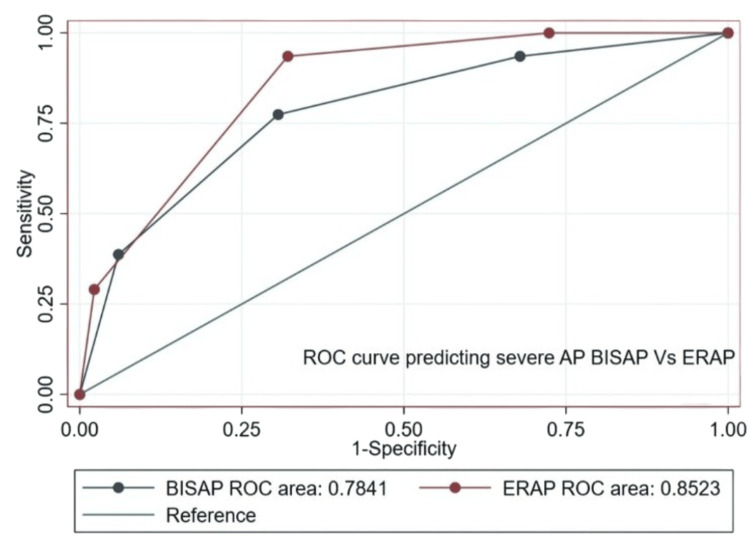
Comparative ROC curves of BISAP and ERAP scores for predicting severe AP. AP: acute pancreatitis; BISAP: Bedside Index for Severity in Acute Pancreatitis; ERAP: Emergency Room Assessment of Acute Pancreatitis; ROC: receiver operating characteristic.

**Figure 3:**
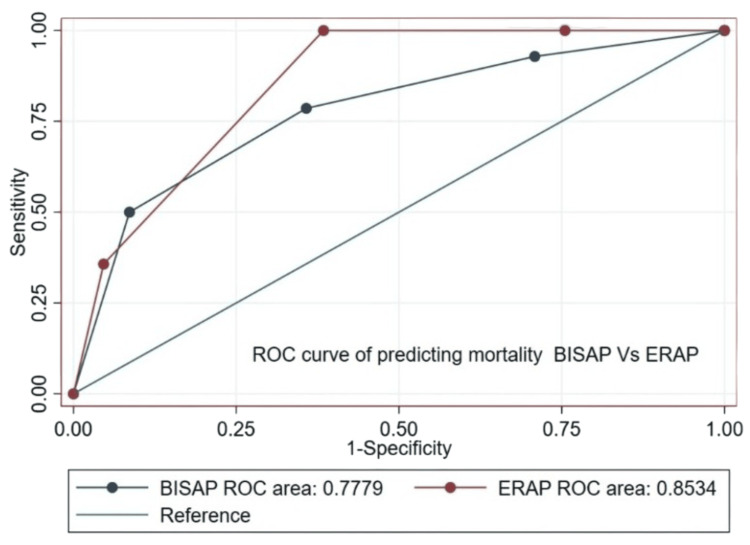
Comparative ROC curves of BISAP and ERAP scores for predicting mortality. BISAP: Bedside Index for Severity in Acute Pancreatitis; ERAP: Emergency Room Assessment of Acute Pancreatitis; ROC: receiver operating characteristic.

The AUC for ERAP in predicting severe AP was 0.852 (95% CI 0.797-0.908) compared to 0.784 (95% CI 0.695-0.873) for BISAP. For mortality, the ERAP AUC was 0.853 (95% CI 0.793-0.913) vs. 0.778 (95% CI 0.643-0.913) for BISAP. For organ failure, the ERAP AUC was 0.843 (95% CI 0.784-0.903) compared to 0.844 (95% CI 0.776-0.912) for BISAP. ERAP showed numerically higher predictive accuracy for severe AP (P = 0.1190) and mortality (P = 0.2829), though not statistically significant (Figures 2, 3).

**Figure 4:**
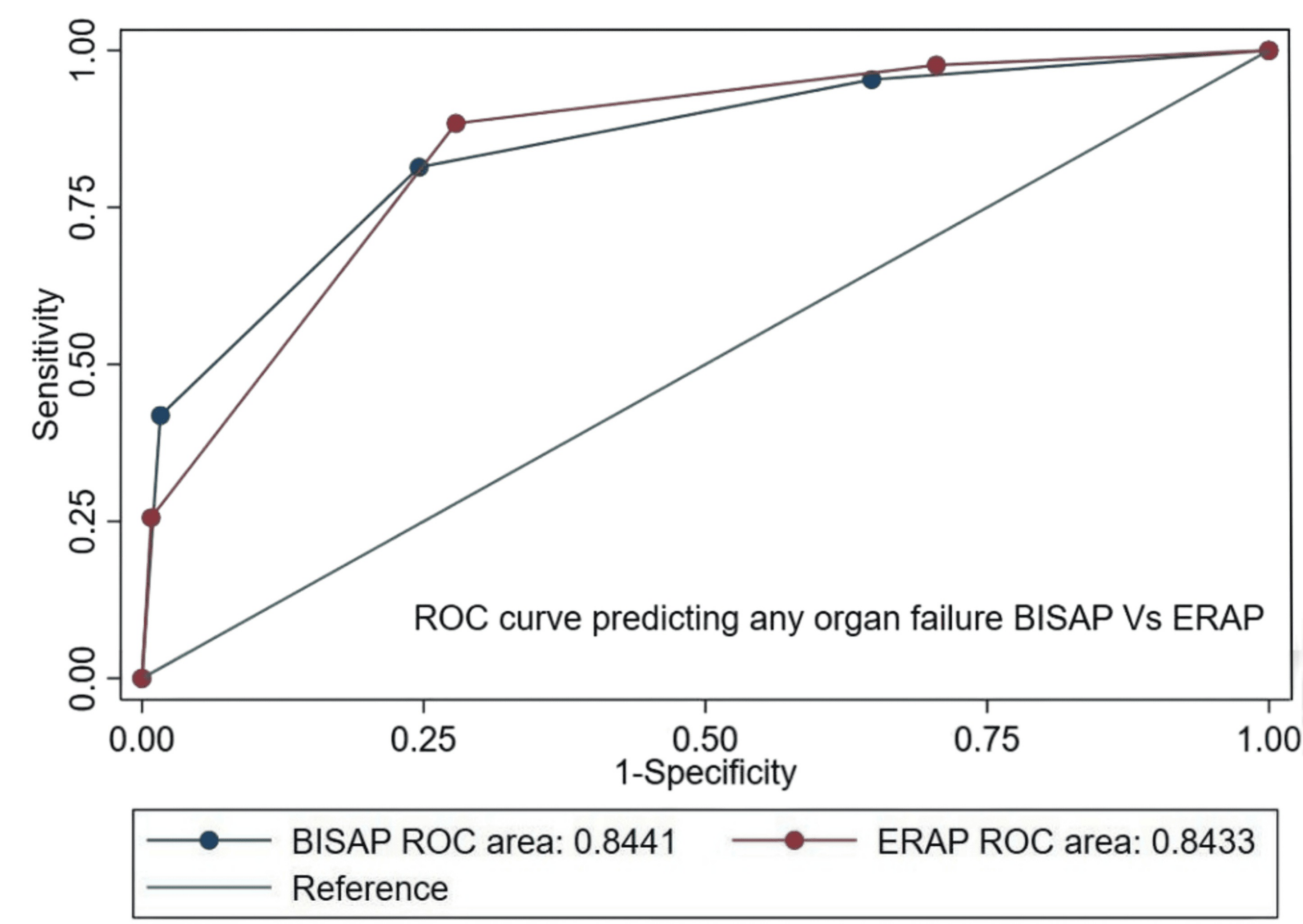
Comparative ROC curves of BISAP and ERAP scores for predicting any organ failure. BISAP: Bedside Index for Severity in Acute Pancreatitis; ERAP: Emergency Room Assessment of Acute Pancreatitis; ROC: receiver operating characteristic.

For any organ failure, both scores performed similarly (Figure 4), with no significant difference observed (P = 0.983).

## Discussion

In this prospective observational study, ERAP and BISAP scores were evaluated for the early prediction of severe AP, mortality, and organ failure in the ED setting. BISAP, a widely used score, continued to perform reliably for predicting severe AP, while ERAP demonstrated slightly higher but statistically insignificant predictive accuracy.

The BISAP score was extensively studied and is one of the easiest scores available in the prognostication of AP. Our study confirmed that BISAP remains a reliable tool for severe AP, consistent with prior studies [11,12].

Compared with earlier studies, the ERAP score in our cohort showed an AUC of 0.852 for predicting severe AP, higher than the 0.689 reported by Rasch et al. [8]. This variation may be explained by differences in patient selection, severity proportion, and diagnostic criteria. For mortality prediction, the ERAP AUC of 0.853 was consistent with the 0.887 reported by Rasch et al. [8], indicating reproducible performance across diverse populations.

The first prospective validation of ERAP by Phan et al. [13] in a Vietnamese cohort demonstrated a statistically significant advantage for predicting severe AP. In contrast, the lack of significance in our study may be due to a lower proportion of severe AP (18.8% vs. 33.5%), which reduced statistical power. Additionally, demographic and etiological variations, such as a younger mean age (37.9 years) and predominance of alcohol-related AP, may have influenced overall severity and score performance. The relatively lower BISAP performance in our cohort might reflect the younger population, minimizing the influence of age > 60 years, a BISAP variable.

The strengths of this study include its prospective design, standardized scoring using Revised Atlanta Criteria 2012, and direct comparison of ERAP and BISAP. Importantly, it provides one of the earliest prospective validations of ERAP in an Indian population, supporting its potential value in emergency settings.

This study has several limitations. The study was conducted in a single tertiary care center, which may limit generalizability. Although demographic details and relevant comorbidities were recorded at admission, comorbidity-based outcome prediction was not separately analyzed. The relatively low proportion of severe AP may have limited the statistical power to detect significant differences between the two scoring systems. Long-term post-discharge outcomes were not evaluated. Multicenter studies with larger sample sizes and extended follow-up are required for broader validation.

## Conclusions

The ERAP score emerges as a simple, rapid, and reliable bedside tool for early risk stratification of AP in the emergency department. Although it showed only marginally higher predictive accuracy than BISAP, its ease of calculation makes it practical for emergency physicians. ERAP’s high NPV allows clinicians to identify low-risk patients confidently, optimize monitoring intensity, and allocate resources efficiently. Incorporating ERAP into early evaluation protocols may enhance triage and patient outcomes. Future multicenter studies are encouraged to validate these findings and explore ERAP’s integration with other predictive systems for comprehensive AP management.
